# Effect of Soil pH on the Growth, Reproductive Investment and Pollen Allergenicity of *Ambrosia artemisiifolia* L.

**DOI:** 10.3389/fpls.2018.01335

**Published:** 2018-09-20

**Authors:** Rodolfo Gentili, Roberto Ambrosini, Chiara Montagnani, Sarah Caronni, Sandra Citterio

**Affiliations:** ^1^Department of Earth and Environmental Sciences, University of Milano-Bicocca, Milan, Italy; ^2^Department of Environmental Science and Policy, University of Milan, Milan, Italy

**Keywords:** weed control, weed management, agriculture area, allergic reaction, health risk, liming

## Abstract

Despite the importance of soil reaction for alien plant establishment, few and incomplete studies have included this key factor so far. In this study, we investigated the effects of soil pH on the germination, growth (plant height, width, dry weight, etc.) and reproductive investment (inflorescence size and n° of flowers) of *Ambrosia artemisiifolia* (common ragweed), an allergenic species that is highly invasive and alien in Europe, through a replicated experiment in controlled conditions. In addition, we determined if soil pH has an effect on the total pollen allergenicity of the species. After preliminary germination tests on agar at different pH (from pH4 to pH8), plants were grown in natural soils with pH values of 5 (acid), 6 (sub-acid) and 7 (neutral) obtained by modifying a natural soil by liming methods (calcium hydroxide solution). Results showed that plants grown at pH7 were shorter and developed leaves at a slower rate than those grown at pH5 and pH6; plants grown at pH7 did not produce flowers and pollen. We also observed that, at pH5 and pH6, larger plants (as assessed by the dry weight of the aerial biomass) had both larger and more numerous inflorescences and emitted pollen earlier. Finally, the IgE-binding signal was higher in pollen samples collected from plants grown at pH5 (Integrated Optical Density, IOD, range: 1.12–1.25) than in those grown at pH6 (IOD range: 0.86 −1.03). Although we acknowledge the limitations of only testing the effects of pH in controlled conditions, this study suggests that soil pH greatly affects the growth and development of *A. artemisiifolia* and indicates that it may have a role in limiting the distribution and hazardousness of this plant. Future field tests should therefore assess the effectiveness of liming in the management and control of ragweed and other alien species.

## Introduction

Invasive Alien Plants (IAPs) have a great impact on the structure and function of ecosystems. A growing body of literature has shown that various invasive plants decrease local plant species diversity, increase ecosystem productivity, alter the rate of nutrient cycling, affect human health and therefore affect ecosystem services and human well-being (Richardson and van Wilgen, 2004; Pejchar and Mooney, 2009; Ehrenfeld, 2010; Vilá et al., 2011; Mannino and Balistreri, 2018). The characterization of IAPs and the study of the environmental factors underlying their success are therefore pivotal to develop effective IAP control measures.

Beyond climate, soil characteristics are believed to play an important role in the survival and performance of alien plants and therefore in successful invasion (Caplan and Yeakley, 2006). Soil reaction (pH), in particular, can be considered a key variable due to its influence on many other soil proprieties and processes affecting plant growth. Indeed, microorganism activity as well as nutrients solubility and availability are some of the most important processes that depend on pH. For instance, in acid soils, most micronutrients are more available to plants than in neutral-alkaline soils, generally favoring plant growth (Lončarić et al., 2008). However, some of these micronutrients, along with non-essential elements, can become toxic when their concentration is too large. In contrast, in alkaline soils, although the availability of most macronutrients is increased, phosphorus and micronutrient availability is generally reduced and their lower levels can adversely affect plant growth. Specifically, many plant characteristics (i.e., traits) such as height, lateral spread, biomass, flower size and number, pollen production, etc., are influenced by pH (Jiang et al., 2017).

IAPs usually possess broader tolerance to environmental conditions, including pH (Dassonville et al., 2008; Hao et al., 2017), than crop and native plants, which have an optimum for pH mostly ranging from 5.5 to 6.5 (Islam et al., 1980; Köpp et al., 2011). This characteristic allows them to adapt to a great variety of soil types and thus to spread vigorously, also colonizing environments not suitable for native species (Sǎrǎteanu et al., 2010). Despite the tolerance of some weeds to different pH having been reported, especially in agriculture, the impact of different soil pH on IAPs has been seldom studied so far (Gilbert and Lechowicz, 2005; Caplan and Yeakley, 2006; Zeng and Clark, 2013).

Among IAPs, *Ambrosia artemisiifolia* L. (common ragweed) is a species of great concern in Europe. Since the nineteenth century, this species of North American origin has been accidentally introduced in Europe where it has naturalized and is now considered an increasingly serious threat to both environment and human health (Montagnani et al., 2017). It is a fast-growing annual weed in crop fields and a colonizer in open-disturbed areas, capable of producing considerable aboveground biomass at various pure stand densities (Patracchini et al., 2011; Fenesi and Botta-Dukát, 2012; Gentili et al., 2017). It also produces large amount of highly allergenic pollen, which represents one of the main causes of pollinosis in many regions of the world (Smith et al., 2013). As for other IAPs, many factors contribute to the increasing spreading of the common ragweed. In particular, since it is a plant that mainly colonizes bare and disturbed soils, especially agriculture areas, abiotic factors related to the characteristics of soil can highly influence its distribution, particularly, soil pH, whose general importance for plant establishment, growth and maturation (i.e., reproductive potential and pollen production) has been largely acknowledged, but whose specific effects on *A. artemisiifolia* (and other species) have been poorly investigated to date. In controlled conditions, Sang et al. (2011) demonstrated that *A. artemisiifolia* germination success exceeded 48% in solutions with pH values between 4 and 12, with maximum rates occurring in distilled water at pH 5.57. In addition, in a field study, Pinke et al. (2011) found that the highest common ragweed cover at the edge of Hungarian sunflower fields occurred where the soil was acid (around pH5). In contrast, mainly based on observations conducted in Austria, Essl et al. (2015) reported that *A. artemisiifolia* grows better in moderately alkaline conditions, according to Ellenberg indicator values. Similarly, Pignatti et al. (2005), indicated that in Italy the species has a wide ecological amplitude for soil pH measured with the Ellenberg and Landolt indicator values for soil reaction. However, up to now, all these studies focused mainly on the distribution of the species in areas with different pH, and no information was available on the influence of soil pH on its growth and reproductive performances. Recently, some farmers (i.e., Bottega Agricola Fratelli Airoldi, Lombardy Region, Italy) who work on croplands highly invaded by *A. artemisiifoila*, observed that the addition of calcium hydroxide to the soil for improving its characteristics and increase its pH value, also inhibits the growth of *A. artemisiifolia*. For all these reasons, although there may be other important environmental factors affecting the fitness of *A. artemisiifolia*, we focused our research on soil reaction in order to understand its specific contribution. Hence it is possible to hypothesize that soil pH affects not only the distribution, but also the growth and the reproductive performance of this species, and can be used to control its spread. In addition, studies have demonstrated that also pollen allergenicity is strongly affected by environmental conditions (i.e., changes in temperature, relative humidity and light) during plant growth and flowering (Goto et al., 2004; Ghiani et al., 2016). Hence, it is possible to hypothesize that soil pH may also affect pollen allegenicity of *A. artemisiifoila*, but to the best of our knowledge, no investigation regarding the role of pH on this trait is currently available in the literature.

In this study, we aimed to investigate how pH affects germination, growth-related traits, reproductive investment, pollen production and allergenicity of *A. artemisiifolia*. We grew plants in controlled conditions in a replicated experimental design and used prediction models to accurately estimate the species performance in relation to pH values (Mohebbi and Mahler, 1989; Robson, 1989; Kidd and Proctor, 2001; Hao et al., 2017). In particular, we used nonlinear models following a sigmoid pattern (i.e., logistic curves) to determine how soil pH affects the germination, growth rate of vegetative traits and reproductive investment of *A. artemisiifolia* (Yin et al., 2003; Sun and Frelich, 2011; Paine et al., 2012; Chen et al., 2014) and if soil pH has an effect on the pollen allergenicity of the species.

## Materials and methods

### Plant material and preliminary germination test

All the experiments were conducted using *A. artemisiifolia* seeds collected in a ruderal area near the town of Brescia (northern Italy; N: 45°29′23″; E: 10°11′47″). Seeds were cold-stratified at 4°C for 3 months to overcome seed dormancy and then planted in a tray containing autoclaved natural soil.

Preliminary germination tests were performed at different pH values in 1% plant agar: pH3, pH4, pH5, pH6, pH7, pH8, and pH9 were considered. The pH values were adjusted with 1 mol/L HCl (low values) and NaOH (high values) according to Sang et al. (2011). Before the test, seeds were sterilized in a solution of distilled water added with sodium hypochlorite (NaClO) at 3% for 2 min; seeds were then washed and dried. For each pH, three Petri dishes containing 30 seeds were set up. To prevent contamination and water loss, Petri dishes were sealed with Parafilm. The three replicates of the germination plates for each pH were put in growth chambers (model Sanyo MLR-350; Sanyo Electric Co., Japan), in controlled condition of light (12 h dark/12 h light, 150 μmol m^−2^ s^−1^) for 30 days at a temperature of 25°C. Petri dishes were checked under a binocular microscope for germination weekly and seeds were recorded as germinated once the radicle protrusion occurred. At the end of the germination test, a cutting test was carried out on non-germinated seeds to assess the presence of the embryo. Calculations of the final germination ratio did not include non-germinated empty seeds.

### Soil preparation and plant growth

Based on the effect of different soil pH on the growth of vegetative traits, reproductive investment and pollen allergenicity of *A. artemisiifolia* was tested in pots in growth chambers under controlled conditions of light (12 h dark/12 h light, 150 μmol m^−2^s^−1^; humidity: 65%) at 25°C for 3 months and half (104 growing days), from 9th April to 22th July, 2015. A randomized complete block design experiment with five replicates (number of individuals was limited by the limited space inside the growth chamber) was carried out to examine the effect of soil pH on plant growth.

To reproduce optimal abiotic environmental conditions for the species germination and growth, plantlets germinated in natural soil were transferred into plastic pots (2,000 ml capacity) filled with the same natural soil subsequently arranged at different pH values. Particularly, we used a soil stock collected from an agriculture area highly invaded by ragweed, at Busto Arsizio (Varese, Italy; N: 45°35′59″; E: 8°52′29″), in February 2015. A soil sub-sample was subjected to a physico-chemical characterization at the soil laboratory of the Milan-Bicocca University (see Supplementary Material S1).

We prepared three different soils for plant growth at the optimal pH values selected after the preliminary germination tests on agar: pH5 (acid), pH6 (sub-acid), and pH7 (neutral). To obtain the selected pH values from the natural soil, a liming method was used according to literature (Brown et al., 2008; Thompson et al., 2016). Particularly, calcium hydroxide solutions [Ca(OH)_2_] was added to the natural soil stock, with a pH value of 5.0, to get two amended soils at pH6 and pH7 (see Supplementary Material S2).

During the whole growth period of plants, the pH value of the prepared soils was measured and monitored weekly (see Supplementary Material S3). In order to avoid disturbance to the growing plant, we collected 10 g of soil in the most lateral part of the pot with a small spoon, after moving the superficial part the soil (about 1 cm). The pH of the soil was potentiometrically measured in the supernatant suspension of a 1:2.5 soil:liquid mixture using the pH-meter, model Eutech pH 700 (Eutech instruments); both distilled water and neutral salt (KCl) solution were used: (a) 10.0 g of fine earth (>2 mm) were added with 25 ml of demineralized water or 1 M solution of KCl in a 50 ml beaker; (b) the soil/water suspension was shacked with a glass rod and leave to rest and decant for at least 2 h; (c) the pH electrode was immersed in the clear part of the suspension and the pH value read after stabilization of the measurement.

### Vegetative and reproductive traits

We collected weekly data on vegetative and reproductive plant traits on the five plants grown in each growth chambers:

- plant height (cm), measured from the plant crown to the maximum growing point of the main branch;
- lateral spread (cm), measured as the maximum diameter of the plant;
- maximum leaf length (cm), measured from the petiole base to the leaf apex;
- maximum leaf width (cm);
- presence or absence of floral buds;
- number of male racemes (the spikes with male flower heads);
- male raceme length (cm; measured at the end of anthesis);
- pollen release, monitored as presence or absence during time;
- dry weight of aerial biomass (g), measured at the end of the growth period.

### Pollen collection and allergenicity

Mature pollen of each plant was recovered in transparent collectors, by covering three male inflorescences (when present) with a modified ARACON system (Lommen et al., 2017) until 10 weeks after the start of the treatments.

Slot blot technique was applied to assess the whole allergenicity of pollen collected from the different racemes, using commercial certified pollen of *A. artemisiifolia* (Allergon®) as standard. Soluble protein extracts were prepared according to Aina et al. (2010). Equal volumes of these extracts, containing an identical amount of proteins, were bound to nitrocellulose membrane and first stained with Ponceau S staining solution [0.1% (w/v) Ponceau S in 5% (v/v) acetic acid] to assess the amount of proteins loaded in each well. Membranes were then used to evaluate the immunoreactivity of the different pollen extracts to a pool of sera from ragweed allergic patients, previously selected (Asero et al., 2014). Image analysis was applied to quantify reactivity signals. The integrated optical density (IOD) of immunoreactive spots with respect to the IOD of standard (Allergon®) was measured. At least three different samples for each racemes were analyzed.

### Statistical methods

The final ratio of germinated seeds between each pH was compared using analysis of variance (ANOVA) test, followed by *post-hoc* Tukey multiple comparison test.

We used the FlexParamCurve v. 1.5–3 (Oswald et al., 2012) package in R 3.3.2 (R Core Team, 2016) for modeling growth trajectories of common ragweed. This package provides tools that facilitate fitting parametric curves in nonlinear modes, which is computationally efficient and allows the estimation of parameters of biological significance even on relatively small datasets (Oswald et al., 2012). Despite the package is designed to fit a large family of growth curves, including non-monotonic ones, we parameterized the model for fitting the four-parameter generalized logistic curve (simply “logistic curves” hereafter) by setting *modno* = 12 in the *SSposnegRichards* procedure.

In detail, the four-parameter generalized logistic curve is described by the equation

(1) $$\begin{array}{r} y=\frac{A}{{\left[1+me^{-k\left(t-i\right)}\right]}^{\frac{1}{m}}} \end{array}$$

Where *y* is the estimated value at time *t, A* is the asymptote of the growth trajectory, *k* is the rate at which the slope of the curve changes with time, *i* is the inflection point, corresponding to the time at which the growth is fastest, and *m* is the shape parameter of the generalized logistic curve.

We used the *nlme* procedure in the *nlme* package (Bates, 2005) to fit nonlinear mixed modes (NLMMs) for investigating whether values of the *A, k, i*, and *m* parameters of the curve were affected by soil pH. NLMMs allow for a large flexibility in the parameterization of both the fixed and the random part of the model, but this flexibility also makes it hard to assess the optimal structure of the model. Following a similar procedure described in Sicurella et al. (2014) and Morganti et al. (2017), we therefore ran preliminary analyses to assess which parameter(s) showed variability according to soil pH (entered as a three-level factor), and which parameter(s) showed large among-individual variability, and should therefore be included as random effect(s). These preliminary analyses were run by first interpolating logistic curves to data of each plant separately and noting the estimated value of individual parameters. Then, we used ANOVA models, corrected for inhomogeneity of variance whenever necessary, to test for variation in each parameter according to pH. When these analyses revealed significant variation, the effect of pH was maintained in the final NLMM, while it was excluded otherwise. For instance, ANOVA models showed that parameters *A, k* and *i* from individual plant height model significantly varied among pH levels, while *m* parameter did not. Thus, the NLMM allowed *A, k* and *i* to vary with pH, but not *m*. We also plotted the range of parameters from curves fitted to individual plants and, in the NLMMs, allowed for random variation in those parameters which, at a visual inspection, showed large heterogeneity (see also Sicurella et al., 2014; Morganti et al., 2017). Finally, we controlled for heteroscedasticity by assuming a variation of the variance with time according to an exponential function, as suggested in Oswald et al. (2012) in all models except for that of leaf width because a model assuming homoscedasticity had a lower AICc value, indicating a better fit (details not shown).

Differences in plant dry weight, number of inflorescences, inflorescence size (including only plants that did produce inflorescences), and time to pollen emission between plants grown at different pH were tested in ANOVA models, generalized linear models assuming a Poisson distribution and corrected for overdispersion, linear mixed models and parametric survival regression models assuming a negative exponential distribution (Kleinbaum and Klein, 2005). Plant dry weight was entered as a covariate in all the other models. Plant identity was included as a random grouping factor in the mixed model of inflorescence size to account for repeated measures taken on the same plant. This model was also corrected for heteroscedasticity by assuming that variance increased exponentially with plant dry weight. Time to pollen emission was considered an interval censored variable (Kleinbaum and Klein, 2005) as pollen production was evaluated only during periodic visits. In detail, no plant emitted pollen before 12/6, while some did not produce pollen before the end of the experiment (15/7). Intervals where therefore considered right censored, but not left censored (Kleinbaum and Klein, 2005).

Analyses where run in R 3.3.2 (R Core Team, 2016) with the “nlme” (Pinheiro and Bates, 1995), “survreg” (Therneau and Grambsch, 2000), and “multcomp” (Hothorn et al., 2008) libraries.

## Results

### Germination test

**Figure 1:**
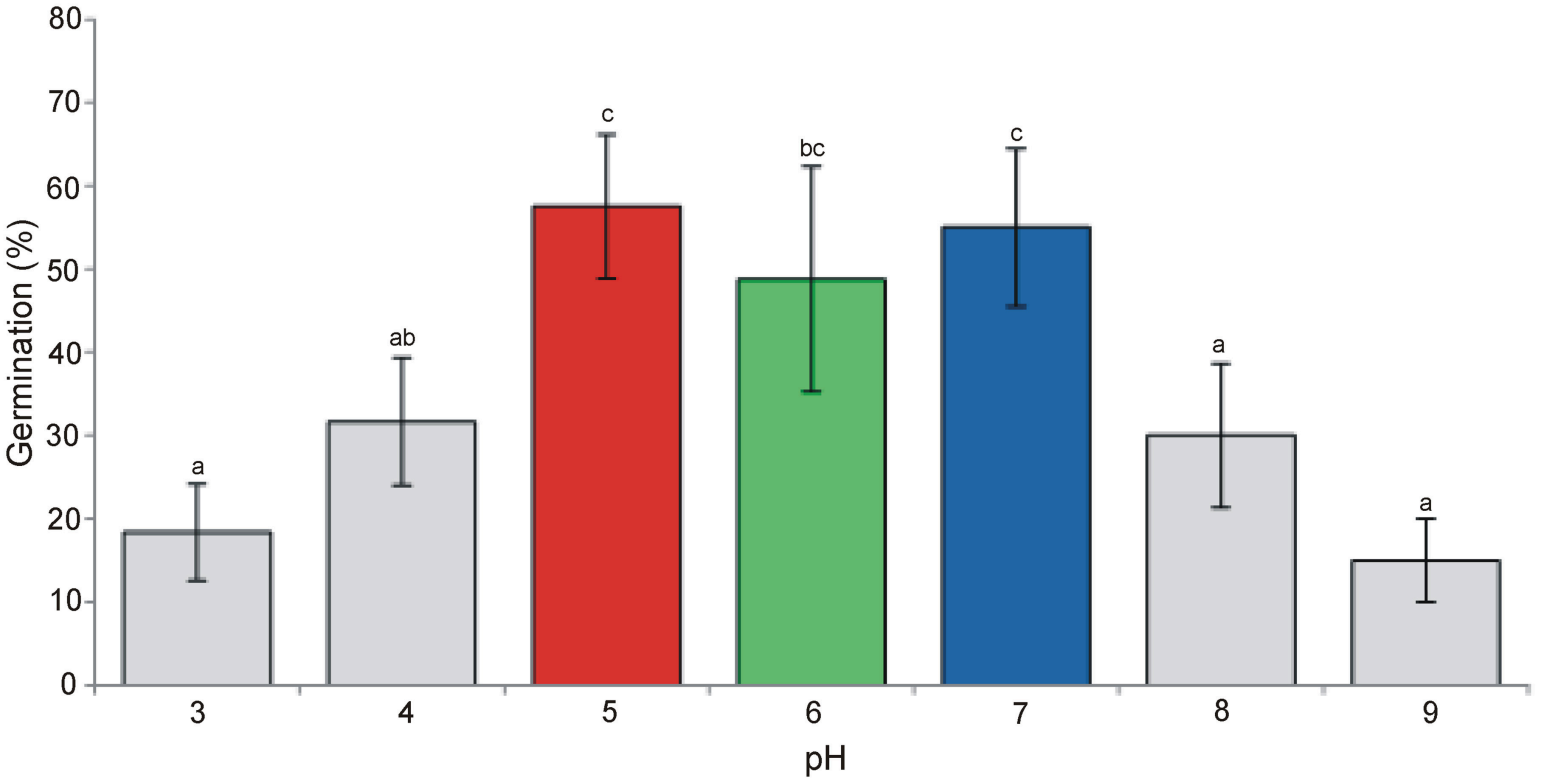
Germination percentage (means + st. dev.) of *A. artemisiifolia* under different pH values (ANOVA: *F* = 11.69; *df* = 6,26; *P* < 0.001). Different letters indicate significant differences of germination at *P* < 0.05 level (Tukey multiple comparison test).

Under the simulated conditions significant differences in the germination percentage were observed between the tested pH values. As a general rule, pH5 (acid), pH6 (sub-acid), and pH7 (neutral) performed better and exhibited germination ratios above (or around) 50% (Figure 1). These values of pH were retained for further analyses regarding the growth rate of plant traits and allergenicity.

### Growth rate and plant traits

We collected 11 measures for 15 plants. One plant, grown at pH7, lost leaves before the last measure, thus we could not measure lateral spread, leaf length and width, but we measured height because the plant was still alive. Sample size is therefore 165 measures for plant height and 164 measures for the other parameters.

**Figure 2:**
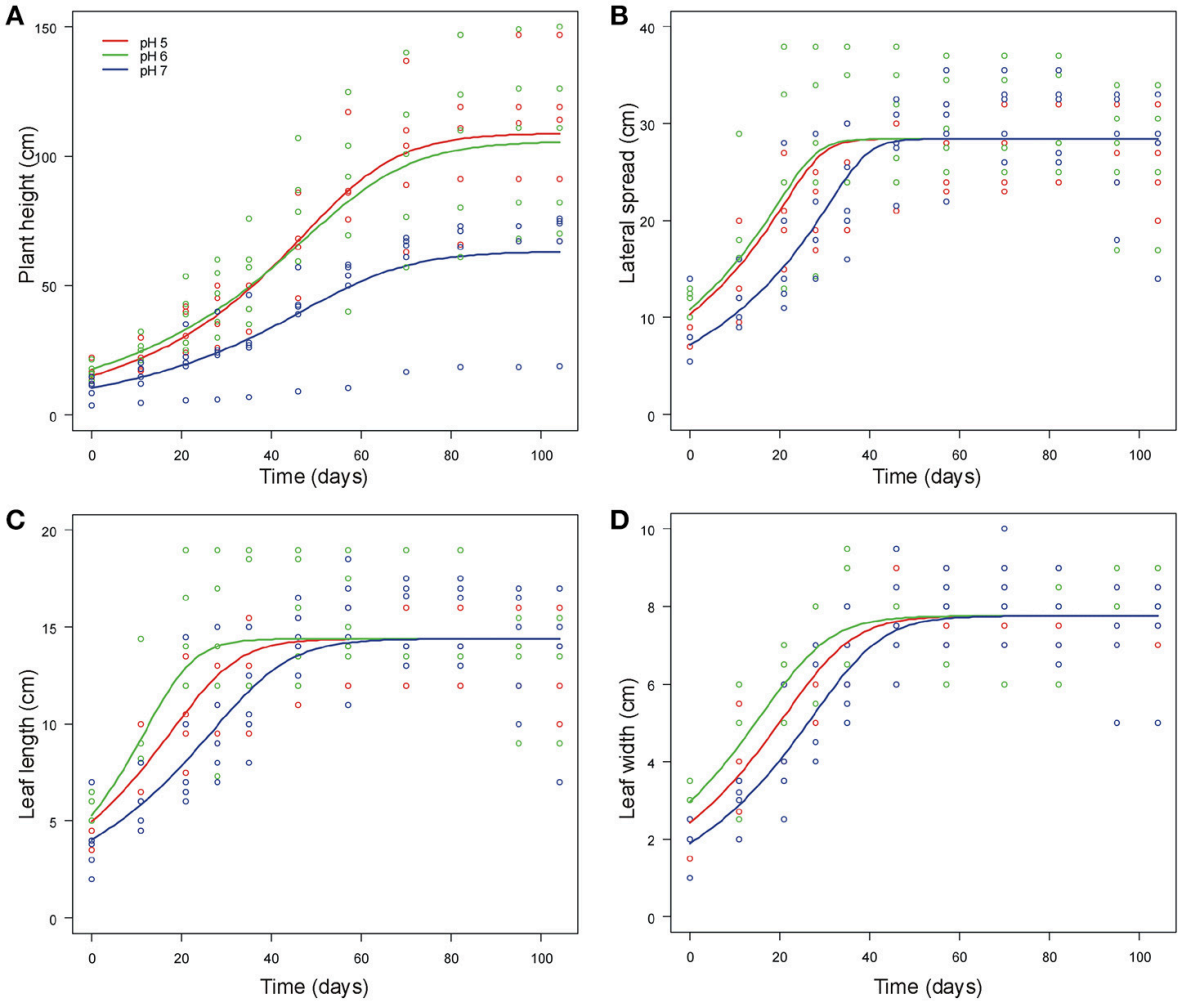
Generalized logistic growth curves of vegetative traits [plant height **(A)**, lateral spread **(B)**, leaf length **(C)** and leaf width **(D)**] of *A. artemisiifolia* according to different pH values (pH5, pH6 and pH7, displayed in different colors) at 25°C.

Final NLMM of common ragweed height indicated that at pH7 plants were shorter than those grown at pH5 and pH6 as suggested by the significant difference in *A* parameters of the generalized logistic curves (Table 1). In addition, plants grew faster at pH5 than at pH6 and pH7 as indicated by the difference in *k* parameters of the growth curves. Overall, growth trajectory of plants grown at pH5 and pH6 were similar, while that of plants grown at pH7 differed (Figure 2A).

**Table 1:** Final NLMM of the growth trajectories.

**Effect**	**Coef**.	**se**	**χ^2^/t**	**df**	***P***
**PLANT HEIGHT**					
*A* × pH			7.042	2	0.030
*k* × pH			9.046	2	0.011
*i*	47.296	1.591	29.733	143	<0.001
*m*	3.244	0.346	9.388	143	<0.001
**LATERAL SPREAD**					
*A*	28.459	1.155	24.642	144	<0.001
*k*	0.324	0.137	2.369	144	0.019
*i* × pH			7.701	2	0.021
*m*	9.021	4.269	2.113	143	0.036
**LEAF LENGTH**					
*A*	14.404	0.677	21.284	142	<0.001
*k* × pH			7.119	2	0.028
*i* × pH			26.288	2	0.021
*m*	4.099	1.988	2.062	142	0.041
**LEAF WIDTH**					
*A*	7.762	0.253	30.712	144	<0.001
*k*	0.158	0.056	2.803	144	0.058
*i* × pH			27.300	2	<0.001
*m*	4.102	2.110	1.944	144	0.054

*Coefficients of each parameter of the generalized logistic curve are shown separately for all parameters that differed significantly among pH levels. Significance of effects that differed among pH levels was assessed by likelihood ratio tests between models that include or exclude the effect under scrutiny. Different letters denote coefficients that differed significantly at post**-**hoc test after Bonferroni correction (P_Bonf_ < 0.05). A: maximum height; k: rate at which the slope of the increasing part of the curve changes with time; i: mean inflection point, m: shape parameter of the generalized logistic curve. Plant identity was included as a random factor in all models. Sample size is 15 plants and 165 measures for plant height and 15 plants and 164 measure for the lateral spread and leaf length and width because one plant lost leaves at the last measure*.

Common ragweed plants grown at different pH showed similar final lateral spread and final size of leaves, as suggested by the fact that *A* parameter did not differ among pH levels in all models. However, leaf development was slower at pH7, as indicated by the fact that *i* parameter, indicating when curves reach the inflection point, was larger for plants grown at pH7 than at lower pH in all models (Table 1, Figures 2B–D). At pH6, however, plant leaves seemed to grow more quickly than at pH5, as indicated by a significantly lower value of *i* parameters of models of leaf length and width (Table 1). In contrast, no significant difference was observed in *i* values of the model of lateral spread (Table 1). Leaves seem also to increase in size at different rates at different pH, as suggested by the significant interaction between *k* parameter and pH. However, *post-hoc* tests could not identify any significant pairwise difference in these parameters after Bonferroni correction (Table 1).

Overall, growth curves showed that common ragweed canopies grew at similar rate at pH5 and pH6. (Table 1, Figure 1B), but more slowly at pH7. Leaves seem to grow fast at pH6, at intermediate rate at pH5 and slow at pH7 (Figures 2C,D).

**Figure 3:**
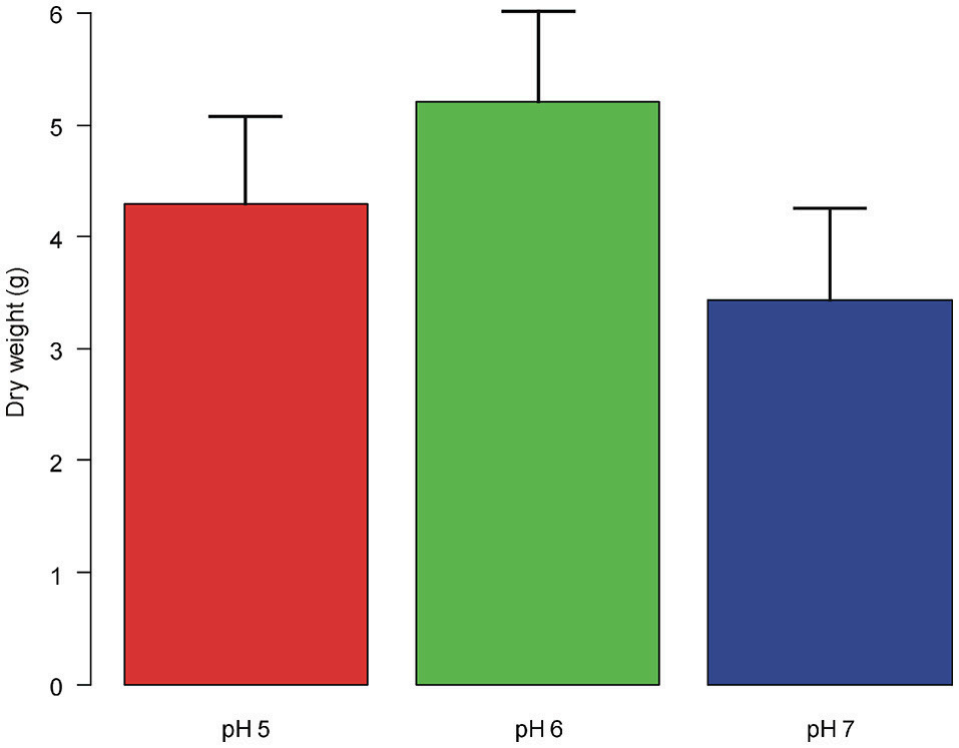
Dry weight of the aerial biomass (means + st. dev.) of *A. artemisiifolia* under different pH values. No significant differences were detected among the treatments.

Plant dry weight did not differ significantly between plants grown at different pH [*F*_(2, 12)_ = 1.213, *P* = 0.331; Figure 3].

### Reproductive investment

**Figure 4:**
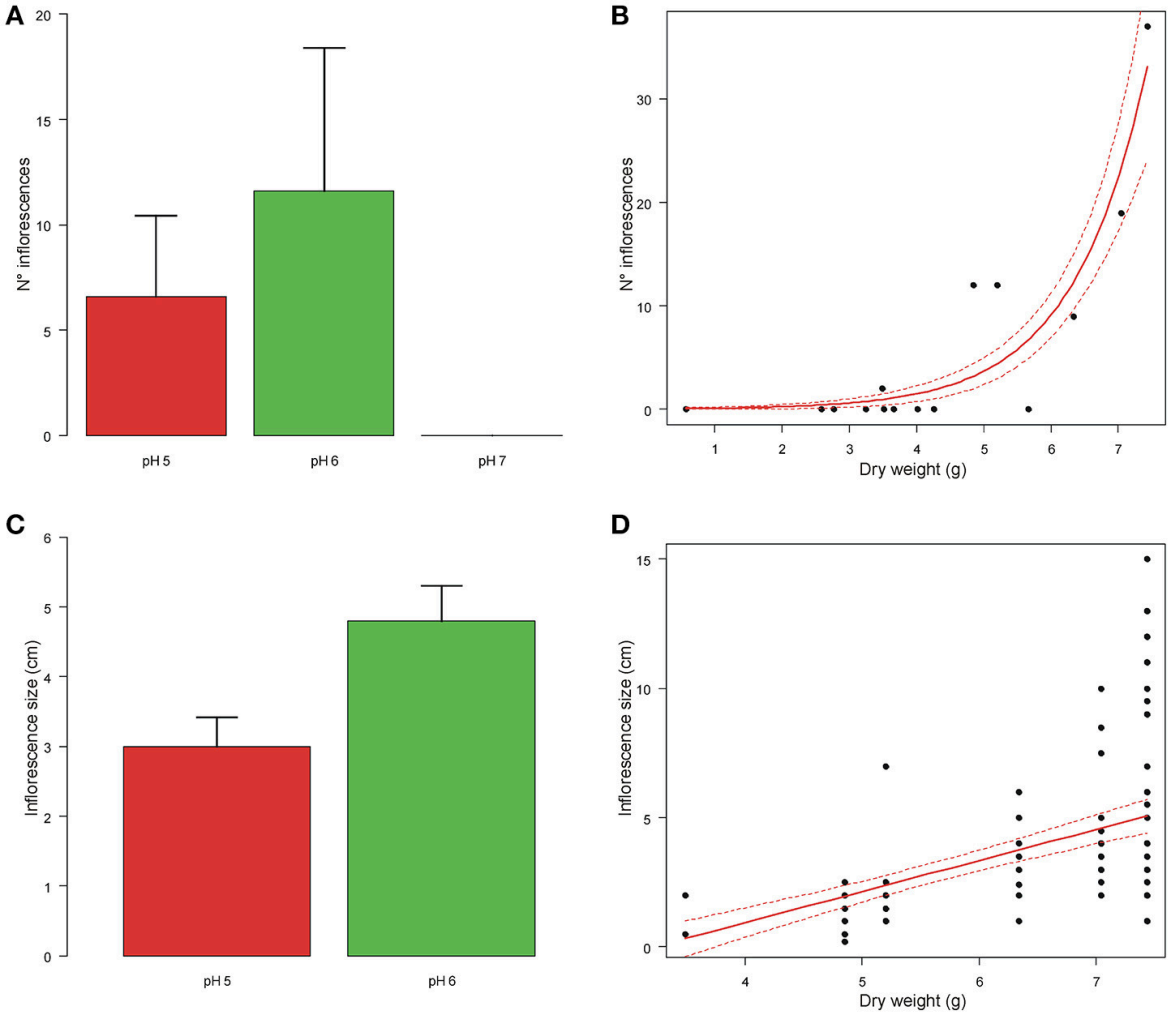
Means number of inflorescences and inflorescence size of *A. artemisiifolia* under different pH values **(A–C)**. At pH7 the plants did not produce any inflorescences. Relationships between number of inflorescences and inflorescence size with dry weight of the aerial biomass **(B–D)**; both parameters increased significantly with dry weight (*P* < 0.001; *P* = 0.047, respectively).

The number of inflorescences increased with plant dry weight (coef = 0.752 ± 0.140 SE, *t* = 5.391, *df* = 11, *P* < 0.001; Figure 4) and differed significantly between plants grown at different pH (*F* = 2.848, *df* = 2,11, *P* = 0.031; Figure 4). This difference was due to the fact that plants grown at pH7 never produced inflorescences, while no difference in the number of inflorescences was found between plants grown at pH5 and pH6 (*F* = 0.003, *df* = 1,7, *P* = 0.958).

Inflorescence size increased with plant dry weight (coef = 1.168 ± 0.358 SE, *t* = 3.266, *df* = 11, *P* = 0.047; Figure 4), but did not differ among plants grown at pH5 and 6 (*t* = 0.215, *df* = 85, *P* = 0.843; Figure 4).

Time to pollen emission decreased with plant dry weight (coef = −0.893 ± 0.330 SE, z = −2.703, *P* = 0.006), but did not differ between plants grown at different pH (log-likelihood ratio test: χ^2^ = 5.379, *df* = 2, *P* = 0.068).

### Pollen total allergenicity

**Figure 5:**
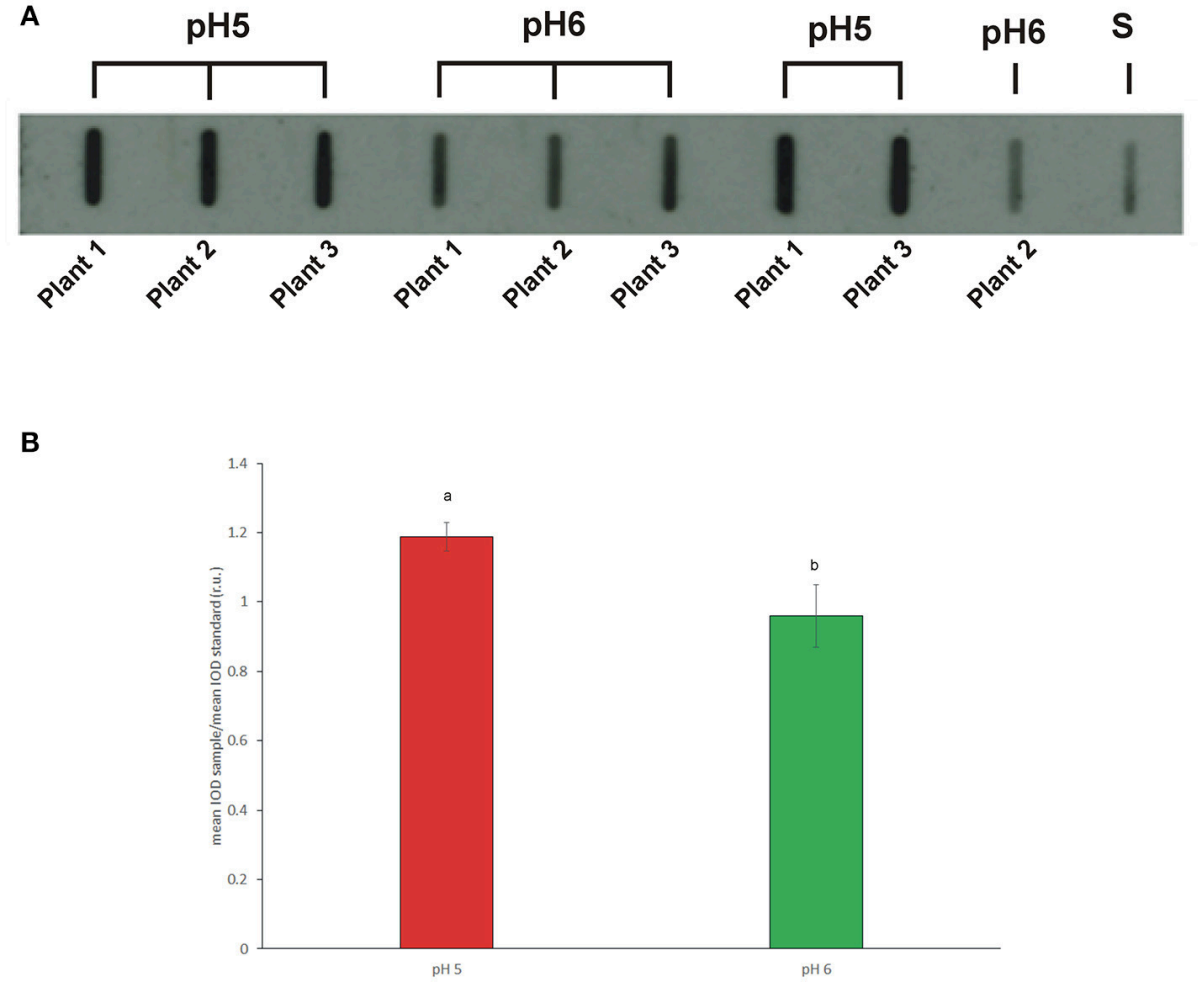
**(A)** Representative slot blot membrane probed with a pool of selected patient sera showing the total allergenicity of pollen samples collected from plants grown in soils at pH5 and pH6. Pollen proteins obtained from single plants by independent extractions were loaded. (S) Standard (protein extract from commercial pollen, Allergon); **(B)** Mean total allergenicity of pollen collected from plants grown at pH5 and pH6. Image analysis was applied to measure the integrated optical density (IOD) of immunoreactive spots with respect to the IOD of the standard (sample IOD/standard IOD). Different letters indicate significant differences between treatments at *P* < 0.05 level.

Pollen from plants grown at pH 5 and 6 was assessed by slot blot technique in order to preserve protein conformation, on which IgE binding may depend. Identical amount of proteins from pollen extracts were bound on a nitrocellulose membrane and subjected to immunoreaction with a sera mix from selected ragweed allergic patients. The Figure 5A shows a representative membrane after immunodetection. Image analysis was applied to quantify immunochemical signals: the integrated optical density (IOD) of immunoreactive sposts with respect to the IOD of standard (sample IOD/standard IOD) was measured. At least three protein extracts from each plant were analyzed and the mean results of five independent experiments were calculated and statistically elaborated (Figure 5B).

On average, all the pollen samples collected from plants grown at pH5 showed a statistically higher IgE-binding signal, ranging from 1.12 to 1.25, than pollen from plants grown at pH6, whose immunochemical signal ranged from 0.86 to 1.03 (*P* < 0.05).

## Discussion

This study demonstrates for the first time that growth and reproductive performances of the IAP *A. artemisiifolia* were greatly affected by soil pH in controlled conditions. Specifically, results confirm that *A. artemisiifolia* is able to germinate and grow in soils with different pH, but its success, in terms of growth of vegetative traits and reproductive investment, increases when the soil reaction is slightly acid, at pH6. On the contrary, the total pollen allergenicity was lower at pH6 than pH5, the only two pH values at which plants produced flowers and then pollen. Since, there is a number of important environmental factors that may control the distribution of common ragweed (i.e., nutrients, temperature, moisture, etc.) and our work only focused on the factor soil pH in controlled conditions, we recognize it may have some limits. Despite this, we would point out that: (a) soil pH is known to control the uptake of macro- and micronutrients (N, Mg, and so on) from soil so it is a quite important factor to be monitored, especially for invasive plants; (b) we used natural soil for growing plants and measuring the pH; this choice made experimental conditions more close to those of field conditions and then the subsequent results useful for future field experiments regarding the species' control; (c) we observed in a crop field (as specified in the introduction) that the amendment of soil using calcium hydroxide highly reduced the species growth.

The method we used in our test to modify the original reaction condition of soil, which implies the addition of calcium hydroxide to increase pH value, can have changed the original quantity of calcium. Thus, calcium could act as confounding factor in understanding the effect of pH on plants. Although calcium is not a major nutrient, it plays a key role in many physiological processes such as the stabilization of cell wall structures, the function of a major secondary-messenger molecule in plants under different developmental cues, the participation in mechanisms of water and nutrient uptake, etc. (White and Broadley, 2003). However, from a static point of view, the soil pH is generally more dependent on elements involved in exchange complex (H^+^ and cations) which buffers possible variations of pH through exchanges between soil and soil solution (Pansu and Gautheyrou, 2003). As a consequence, the addition of calcium hydroxide were intended to reproduce conditions similar to field ones in which calcium is generally the most representative cation in soil exchange complex and to simulate the actual system regulating pH values.

### Germination, growth of vegetative traits, and reproductive investment

The germination rate of *A. artemisiifolia* was higher at pH5 to pH7 than at lower or higher pH values. These results are in accordance with those of Sang et al. (2011) who found the optimum pH of germination between pH5 and pH8. In general, among the intermediate values of pH tested in this study for the subsequent analyses (growth curve and reproductive investment), plants grown at pH5 and pH6 performed better than those grown at pH7.

With regard to vegetative traits, the shortest height as well as the slowest growth rate for all vegetative traits were recorded at pH7. This results are in disagreement with those of an old work of Turner (1928) reporting that *A. artemisiifolia* was more abundant and taller at neutral–slightly alkaline soil (pH 7.0–7.3) than plants grown in sub-acid and acid soils (below pH7). Tessmer et al. (2013) found that slower growth rate correlated with a reduced photosynthetic efficiency in different populations of *Arabidopsis thaliana*. Nevertheless, in our results although *A. artemisiifolia* grown at pH7 exhibited a slower growth for all traits than at pH5 and pH6, the final values of traits related to leaf size (length, width and lateral spread) did not significantly differ among pH values, indicating a great ability of the plant to grow (i.e., adapt), at a slower rate, at less suitable pH conditions (pH7 in our case). Physiological mechanisms of adaptation of plants to non-optimal soil pH are well-known in literature. Particularly, root-induced changes in the rhizosphere occur through the release of charges carried by H^+^ or OH^−^ to balance cation–anion uptake at the soil–root interface (Hinsinger et al., 2003). This behavior is consistent with observations we made in our study, since the pH of soil conspicuously decreased over time at all the pH values monitored (data not shown). However, observing the whole dataset of vegetative plant traits, plants grown at pH7 exhibited the lowest absolute value of biomass (even if not significantly different from pH5 and pH6), in addition to the lowest values of plant height and velocity of growth in addition to the lack of male inflorescences. Likely a trend, not captured by our data, indicating less vigor of the species at pH7 can be invoked and should be taken into account.

In any case, these results should be carefully evaluated considering some confounding factors relating to the soil ecosystem: (a) soil pH is known to influence the availability and uptake of a micronutrient like Mg that is implicated in the plant's photosynthetic efficiency (Dighton and Krumins, 2014). For instance, at high pH, Ca, and Mg tend to form less or not available compounds when reacting with P and many micronutrients (Barber, 1995); (b) complex interactions between biotic (i.e., bacteria and fungi) and abiotic factors that occur within the soil ecosystem.

Key elements of soil useful for plants growth are nitrogen (N), potassium (K) and phosphorous (P) that different plants species can preferentially absorb according to pH. For instance, as regards N content, plants can adsorb it in the forms of ammonium (NH${}_{4}^{+}$) or nitrate (NO${}_{3}^{-}$), according to soil pH (Serna et al., 1992; Abbasi et al., 2017). Since *A. artemisiifolia* is a considered a nitrophilic plant (Qin et al., 2014; Skálová et al., 2015) nitrogen very likely played a major role in influencing the plant growth in our experiment, also considering that the natural soil we used for plants exhibited good concentrations of nitrogen (see Supplementary Material S1). Particularly, our results support the findings of Nádasi and Kazinczi (2011) that *A. artemisiifolia* grows better in sub-acid soils (pH = 5.87) with higher ammonium-nitrate content than in neutral–slightly alkaline soils (pH = 7.26) with lower ammonium-nitrate content. However, Leskovsek et al. (2012) observed that pure stands of *A. artemisiifolia* plants grown in field (at pH 6.6) and greenhouse experiments (in peat moss; pH not indicated), produced considerable biomass and seeds under various densities and nitrogen rate.

Contrary to our observation, in another experiment involving other widespread/ruderal species (i.e., *Alliaria petiolata* and *Sonchus arvensis*) plants were capable of performing better, in terms of biomass, in less acid soils, toward pH7 (Zollinger and Kells, 1991; Anderson and Kelley, 1995) confirming a species-specific behavior of plants for nutrient absorption with respect to soil reaction.

With regards to reproductive investments, at pH6 and pH5 *A. artemisiifolia* plants showed similar trends also in terms of number of inflorescences and inflorescence size while at pH7 they did not produce any inflorescence, confirming the tendency observed for vegetative traits, i.e., at pH7 plant exhibited a worst performance. This behavior of the plants at pH7 could also be due to the effect of an excess of calcium hydroxide after the manipulation of the natural soil we used in our experiment. With regards to time to pollen emission it significantly decreased with plant dry weight (shorter at pH5 than pH6), as expected.

The influence of pH on the reproductive investment has been already observed for other species (no literature information were found for *A. artemisiifolia*). For instance, in a work on the effect of different pH values (from 4.5 to 8) on the vegetative and reproductive growth of Rose cv., the best plant performance, in terms of number of buds, was obtained at pH6.5 and the lowest one at pH8 (Rosta and Rezaei, 2014). In contrast, Lankinen (2000) found that low pH (pH4) had a negative effect on production of *Viola tricolor* flowers and seeds, which decreased of about 18 and 33% with respect to intermediate pH values.

Our results highlight that intermediate/slightly acid values of pH are in general most suitable for the growth and reproduction of *A. artemisiifolia*. Most plant nutrients are known to be optimally available to plants at intermediate/sub-acid pH ranges and are compatible to plant root growth (Jensen, 2010). Plants growing in too acid or too calcareous (i.e., alkaline) soils greatly change their uptake ability of micro- and macronutrients and are constantly exposed to either mineral deficiency or metal toxicity (Ramírez-Rodríguez et al., 2005). Consequently, a non-optimal soil pH condition for a plant can affect its growth and reproductive performances, as we have observed in this study for *A. artemisiifolia* grown at pH7. In any case, also reproductive investment in response to different pH ranges is species-specific as a result of evolutionary history and adaptation ability to environment of each species (Ware, 1990; Zeng and Clark, 2013; Offord et al., 2014).

### Pollen allergenicity

In this work, the soil pH at which a plant was grown affected common ragweed pollen allergenicity, which, in our experimental condition, was lowest at pH6. Unfortunately, no specific studies on the effects of soil pH on pollen allergenicity were performed to date. However, previous studies demonstrated that environmental variability and biotic/abiotic stress led to differences in the amount and type of pollen allergens. For instance, Ghiani et al. (2012) reported an increased allergenicity of pollen of *A. artemisiifolia* populations exposed to road traffic pollution. Climate change was indicated to affect pollen allergenicity determined by a higher concentration of the Amb a 1 allergen in pollen of plants exposed to higher temperatures and drought (El Kelish et al., 2014; Ghiani et al., 2016). Cloutier-Hurteau et al. (2014) measured the transfer of trace elements (i.e., Ba, Cd, Cr, Cu, Mn, etc.) from soil to pollen of *A. artemisiifolia* plants growing in ruderal sites in order to validate the impact of these elements on human health, as possible explanation for the increase of allergy symptoms within industrialized areas. They found positive relationships between the concentration of some trace elements (Cd Ni and Pb) in pollens and in soil or roots. Unfortunately, they did not measure the allergenicity of those pollen grains; moreover they did not find any relation of the trace elements concentration in pollen grain with soil pH probably due to the limited pH range of the investigated soils (7.31–8.39 range) as well as to the high pH values that are unfavorable to element mobility in soils. In our experiment, we can suppose that the addition of calcium hydroxide to soil in order to increase pH from 5 to 6 interfered with pollen allergenicity. Indeed, we noticed a higher amount of flavonoids in pollen extracts from plant grown at pH6 probably produced to face the presence of calcium. This higher amount of secondary metabolites likely affected the IgE binding explaining the lower allergenicity detected for pH 6 pollen.

### Implication for *A. artemisiifolia* management

Despite the fact that our results in controlled conditions indicate better performances of *A. artemisiifolia* in sub-acid soil conditions, the observations concerning the distribution or abundance of the species are inconsistent in field studies. Several authors found the highest abundance of common ragweed in acid soil, toward pH5 (Ujvárosi, 1973; Szigetvári and Benkö, 2008; Pinke et al., 2011; Li et al., 2014). In contrast, other authors found that the presence of *A. artemisiifolia* is better related to neutral moderately alkaline condition, toward pH7 and pH8 (Turner, 1928; Fumanal et al., 2008; Essl et al., 2015). However, it should be noted that in such studies the vegetative vigor and the reproductive performances of the plant in the growth sites were not reported. The inconsistency of field studies on pH preference of weeds has been related to the covariation of pH range with climatic gradients (annual rainfall and temperature; Pinke et al., 2011, 2012). In our study, a neutral soil, obtained after the addition of calcium hydroxide to a natural acid soil, inhibited the emission of flowers (besides the plant height) during the observation period. This result supports field observations by Italian farmers working on croplands highly invaded by *A. artemisiifolia* regarding the inhibiting effect of the addition of calcium hydroxide on the growth of the species.

In the management of IAPs, manipulating the soil attributes is one of the strategies to achieve a successful control, especially in agricultural environments. Particularly, nutrient and soil nitrogen management, highly dependent on soil pH values, or the addition of activate carbon have been used to achieve desired soil properties, and thus plant communities resistant to invasion (Kulmatiski and Beard, 2006; Vasquez et al., 2008). Although we acknowledge the limitations of only testing the effects of soil pH in controlled conditions, our study suggests that further in-field research on the effects of liming on the growth and performances of *A. artemisiifolia* and other IAPs should be carried out, in order to test effective control measures in agricultural environments. Species-specific approaches, may be implemented by applying soil liming methods (that may have management problems and high costs) also tested in combination with other restoration methods (such as N management, plowing, herbicide application, etc.) to enhance resistance of soils and native plant communities to numerous IAPs. In fact, it is surprising that the effect of invasive plants on soil pH has been investigated in numerous circumstances (Ehrenfeld, 2003), but not the opposite.

Interesting findings of our experimental study are that: (a) in not optimal pH conditions (pH7 in our study) *A. artemisiifolia* does not produce buds and inflorescences; in sub-optimal pH conditions for growth (pH5) the length of the pollen emission is reduced compared with the optimal pH conditions (i.e., pH6), even if an opposite pattern was observed for pollen allergenicity. These factors should be considered and may have possible implications during the evaluation of health risk linked to pollinosis.

## Author contributions

RG and SaCi conceived and designed the experiments. CM and SaCa conducted laboratory analyses. RG and RA analyzed the data and wrote results. RG and SaCi wrote the manuscript (Introduction and Discussion); all authors provided editorial advice and revised manuscript.

### Conflict of interest statement

The authors declare that the research was conducted in the absence of any commercial or financial relationships that could be construed as a potential conflict of interest.
