# Supplementary material for: Effect of Soil pH on the Growth, Reproductive Investment and Pollen Allergenicity of Ambrosia artemisiifolia L
Source: Front Plant Sci. 2018 Sep 20;9:1335. doi: 10.3389/fpls.2018.01335 (PMC6158341; doi:10.3389/fpls.2018.01335)

Supplementary Material

Effect of soil pH on the growth, reproductive investment and pollen allergenicity of *Ambrosia artemisiifolia* L.

Rodolfo Gentili*, Roberto Ambrosini, Chiara Montagnani, Sarah Caronni, Sandra Citterio

*** Correspondence:** Corresponding Author: rodolfo.gentili@unimib.it

Supplementary Data

S1. Results of the analysis of the natural soil used for the experiment: chemical (a) and physical (b) characteristics

a) Soil chemical characteristics	
pH H_2O g/kg	5
pHKCl	4,7
Organic C g/kg	30
S.O.	51
N tot g/kg	2,95
C/N	10,1
CSC	11,38
Ca cmol(+)kg	1,45
Mg cmol(+)kg	0,33
K cmol(+)kg	0,03
TBS	15,9
Assimilable P mg/kg	51

b) Soil texture	

Grain-size class	g/Kg
Sg 2-0,1 mm	495
Sm 0,1-0,05 mm	100
S 2-0,05 mm	595
L 0,05-0,0002 mm	321
A 0,0002 mm	84

S.2. Soil liming. Once the chemical and physical analyses were carried out and the pH value of the agricultural soil was determined (pH5), the pH of the soil was adjusted by liming methods in order to obtain two growing soils at pH6 and pH7 respectively.

The soil was first sieved with a wide mesh sieve in order to remove all stones that might affect the soil reaction and then sterilized in an autoclave at 120 °C for 20 min.

A calcium hydroxide solution ($Ca(OH)_2$ was prepared for correcting the pH of the acid soil. In particular, after a series of tests to determine the lime requirement of the used soil, we treated 10 kg of soil with a solution of 10 g $Ca(OH)_2$ in 2.7 L of demineralized water, to obtain a soil at pH6; subsequently we treated 10 kg of soil with a solution of 50 g $Ca(OH)_2$ in 2.7 L of demineralized water to obtain a soil at pH7. The treated soils were left to dry at room temperature for about three weeks. For the determination of the pH of the soils, the pH was measured in aqueous suspension.

S.3. pH monitoring. During the growth period of plants, the pH value of the prepared soils (i.e. pH5, pH6, and pH7) was measured and monitored weekly.

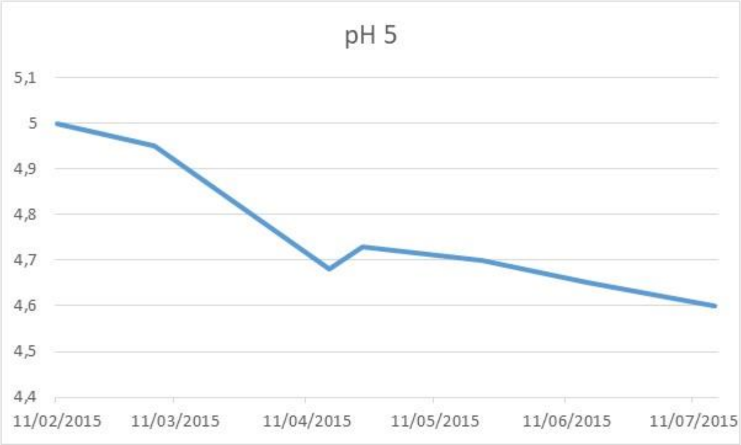

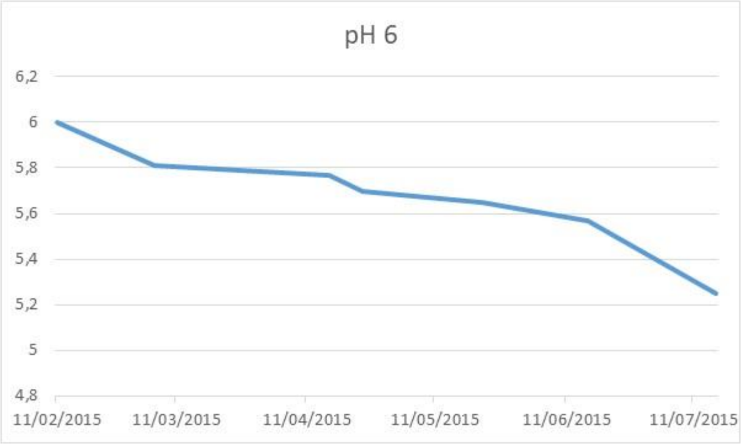

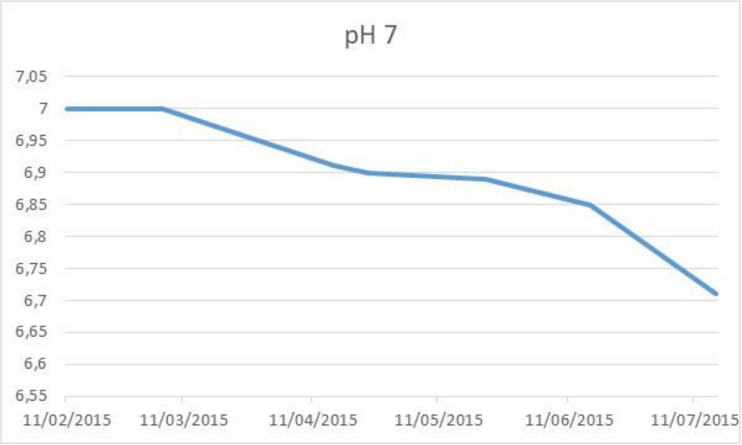

Supplement: Supplementary file 1 [file Data_Sheet_1.PDF]
